# Kinetochore–microtubule attachment is sufficient to satisfy the human spindle assembly checkpoint

**DOI:** 10.1038/ncomms9987

**Published:** 2015-12-01

**Authors:** Banafsheh Etemad, Timo E. F. Kuijt, Geert J. P. L. Kops

**Affiliations:** 1 Hubrecht Institute—KNAW (Royal Netherlands Academy of Arts and Sciences), Uppsalalaan 8, 3584 CT Utrecht, The Netherlands; 2 Molecular Cancer Research, University Medical Center Utrecht, 3584 CG Utrecht, The Netherlands; 3 Center for Molecular Medicine, University Medical Center Utrecht, 3584 CG Utrecht, The Netherlands; 4 Cancer Genomics Netherlands, University Medical Center Utrecht, 3584 CG Utrecht, The Netherlands

## Abstract

The spindle assembly checkpoint (SAC) is a genome surveillance mechanism that protects against aneuploidization. Despite profound progress on understanding mechanisms of its activation, it remains unknown what aspect of chromosome–spindle interactions is monitored by the SAC: kinetochore–microtubule attachment or the force generated by dynamic microtubules that signals stable biorientation of chromosomes? To answer this, we uncoupled these two processes by expressing a non-phosphorylatable version of the main microtubule-binding protein at kinetochores (HEC1-9A), causing stabilization of incorrect kinetochore–microtubule attachments despite persistent activity of the error-correction machinery. The SAC is fully functional in HEC1-9A-expressing cells, yet cells in which chromosomes cannot biorient but are stably attached to microtubules satisfy the SAC and exit mitosis. SAC satisfaction requires neither intra-kinetochore stretching nor dynamic microtubules. Our findings support the hypothesis that in human cells the end-on interactions of microtubules with kinetochores are sufficient to satisfy the SAC without the need for microtubule-based pulling forces.

## Abstract (web summary)

The spindle assembly checkpoint protects against premature chromosome segregation during mitosis but it is not known whether microtubule attachment to the kinetochore, or force generated from this interaction, is being monitored. Here the authors uncouple these processes and show that microtubule attachment is sufficient to satisfy the checkpoint.

## Graphical abstract

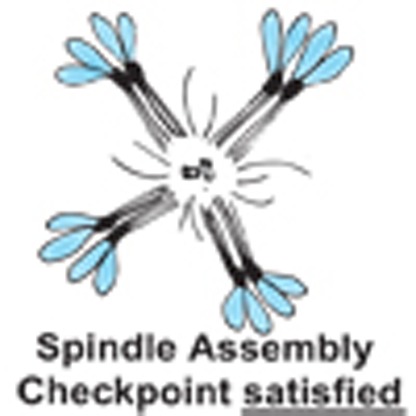

Error-free chromosome segregation in human cells requires prior biorientation of all chromosomes and satisfaction of the spindle assembly checkpoint (SAC; refs 1, 2). Despite profound insights into the molecular mechanisms of SAC signalling gained in recent years^3^, a fundamental question remains unresolved: what defect in spindle assembly is ‘sensed' by the SAC? Lack of kinetochore–microtubule attachment, absence of the force generated by dynamic microtubules that signals stable biorientation of chromosomes, or both? Although various studies have addressed this^4^,^5^,^6^,^7^,^8^,^9^,^10^,^11^,^12^,^13^, a consensus has not been reached^14^,^15^,^16^. This may in part be due to variations in experimental model systems and/or to approaches that have not undisputedly allowed for a way to maintain chromosome–spindle attachments while preventing biorientation, without affecting the SAC machinery. Moreover, distance between sister kinetochores (‘tension') was often used as a proxy for a state of stable biorientation required to satisfy the SAC, but recent findings indicate that this may not be a valid assumption^17^,^18^. These studies have inspired current models that invoke tension within a kinetochore, generated by microtubule-pulling forces, as the signal that satisfies the SAC.

In human cells, iterative rounds of error correction are required to achieve biorientation after kinetochores initially acquire microtubule connections in early prometaphase^19^,^20^. Every round of correction prevents subsistence of non-bioriented kinetochores through microtubule detachment^21^. Non-bioriented but stably attached kinetochores are therefore non-existent in human cells. The kinase Aurora B achieves error correction by decreasing affinity for microtubules of the main microtubule-binding complex KMN (composed of the KNL1, MIS12, and NDC80 subcomplexes) at kinetochores through multi-site phosphorylation^22^. Hampering Aurora B activity through chemical inhibition gives rise to stably attached, non-bioriented kinetochores^23^ and could potentially be used to study whether the SAC is able to ‘sense' lack of biorientation. However, recent evidence of direct Aurora B engagement in SAC signalling renders approaches such as these inconclusive^24^,^25^,^26^,^27^,^28^,^29^. A key target of Aurora B is the HEC1 protein that receives multiple phosphorylations in its N-terminal tail. A non-phosphorylatable HEC1 tail mutant, HEC1-9A, has an increased affinity for microtubules and causes persistent kinetochore–microtubule interactions^30^,^31^,^32^,^33^. We thus reasoned that expression of HEC1-9A would enable the maintenance of stable attachments in the absence of biorientation without affecting kinetochore composition and signalling, and thus provide a tool to understand what state of chromosome–spindle interactions satisfies the SAC. Here, we show that the SAC is satisfied in HEC1-9A-expressing cells with non-bioriented kinetochore–microtubule attachments that lack significant intra-kinetochore stretch. Our findings indicate that stable end-on microtubule attachments are sufficient to silence the SAC.

## Results

### The SAC is satisfied in HEC1-9A cells with monopolar spindles

We used our previously published HEC1 reconstitution system in which green fluorescent protein (GFP)-HEC1 variants are expressed from a conditional promoter in an isogenic background of HeLa-FlpIn cells^34^. This allowed equal expression of RNAi-resistant mutants in a doxycycline-inducible fashion while depleting endogenous HEC1 by short interfering RNA (siRNA; Supplementary Fig. 1a,b). A tail-deletion mutant (HEC1-Δ80) and a tail mutant containing phosphomimetic substitutions of the Aurora B phosphorylation sites (HEC1-9D) were used as controls^35^,^36^. Expression of GFP-HEC1 variants after siRNA-mediated depletion of endogenous HEC1 resulted in equal levels of GFP-HEC1 at kinetochores (Supplementary Fig. 1c,d). As expected, cells expressing the HEC1 variants displayed chromosome alignment defects and segregation errors, phenotypes previously reported by others (Supplementary Fig. 1e,f)^31^,^33^,^35^,^36^,^37^.

**Figure 1:**
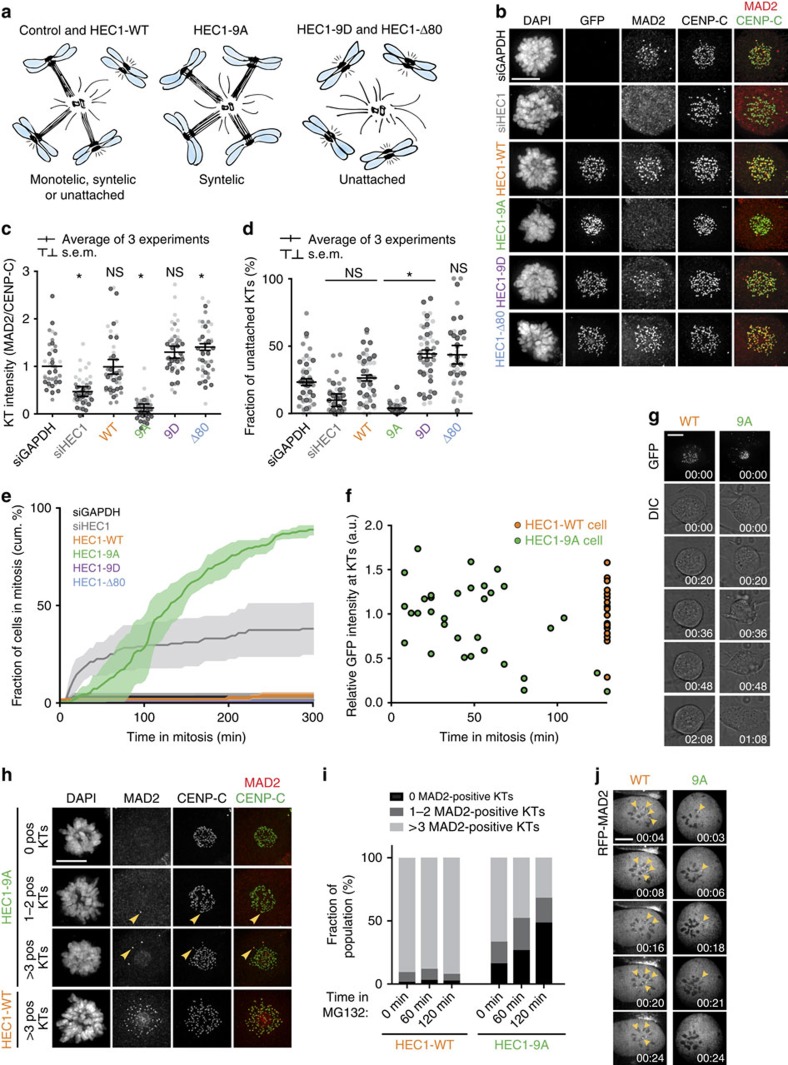
Monopolar HEC1-9A cells satisfy the SAC. (**a**) Cartoons illustrating the experimental set-up that uncouples stable attachments from biorientation. Cells expressing HEC1 variants and forced to be monopolar, as a consequence of treatment with STLC, will have various states of kinetochore–microtubule attachments, as listed below drawings. (**b**,**c**) Immunofluorescent labelling (**b**) and quantification (**c**) of indicated proteins in STLC-arrested cells. Cells were treated with MG132 for 2 h before fixation. Quantification is normalized to the kinetochore (KT) intensity of CENP-C and is the average fold change of three experiments (±s.e.m.) normalized to the values of control cells. Each dot represents one cell. The data points of individual experiments (*n*=49) are depicted in different shades of grey. Quantifications were subjected to unpaired Student's *t*-test against the values measured for control cells. *P<0.01. (**d**) Quantification of the number of MAD2-positive kinetochores as a percentage of the total kinetochores per cell. Conditions and representation as in (**b**). (**e**) Time-lapse analysis of mitotic arrest in STLC-treated cells. Shown is the average of three experiments (solid lines)±s.e.m. (transparent area). (**f**,**g**) Time-lapse single-cell analysis (**f**) and representative stills (**g**) of HEC1-WT and HEC1-9A monopolar cells. For each cell, the total GFP-kinetochore level was measured at mitotic entry, normalized against the average level measured in HEC1-WT cells and plotted in (**f**). Filming started ∼1 h after release from CDK1-inhibitor RO-3306. 21 HEC1-WT, and 33 HEC1-9A cells were followed in two independent experiments. (**h**,**i**) Representative images (**h**) and quantification (**i**) of the number of MAD2-positive kinetochores in HEC1-WT and -9 A monopolar cells in time. Cells entered mitosis in the presence of STLC and subsequently treated with MG132 for the duration of the indicated time. Data are the average of two experiments (*n*=65). Arrowheads in **h** indicate MAD2-positive kinetochores in HEC1-9A cells that point away from the centrosome and move to the periphery or out of the chromatin pack. (**j**) Representative stills from live analysis of HEC1-WT and HEC1-9A-expressing RFP-MAD2. Arrowheads indicate MAD2-positive kinetochores. Filming was started 1 h after release from RO. Scale bars, 10 μm. Scale bars in other panels, 5 μm.

**Figure 2:**
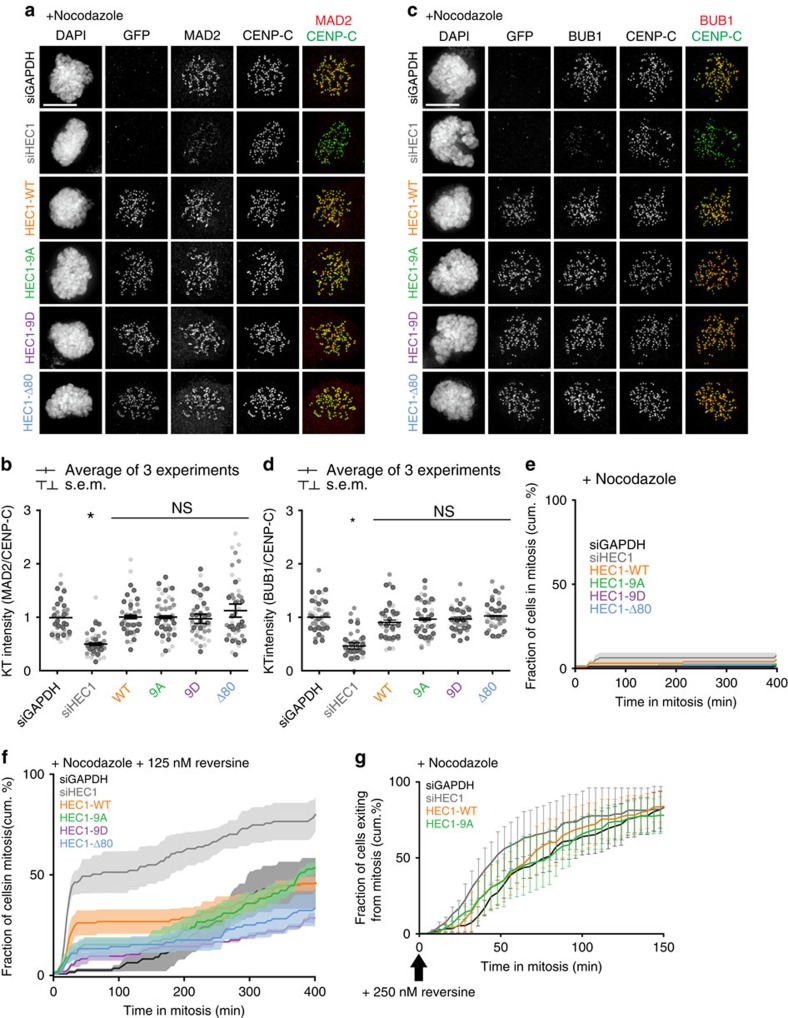
HEC1-9A cells have a fully functional SAC. (**a**–**d**) Immunofluorescent labelling (**a**,**c**) and quantification (**b**,**d**) of indicated proteins in nocodazole-arrested HeLa cells-expressing mutant versions of HEC1. Channel colours of merged images match those of the labels. Quantification is normalized to the kinetochore intensity of CENP-C and is the average fold change of three experiments (± s.e.m.) normalized to the values of siGAPDH-transfected cells. Each dot represents one cell. The data points of three independent experiments are depicted in different shades of grey. A total of at least 47 cells were measured per mutant in (**b**) and 44 in (**d**). For statistical analysis an unpaired Student's *t*-test was performed against the measured values of siGAPDH-transfected cells. *P<0.01. (**e**,**f**) Quantification of time-lapse analysis of duration of mitotic arrest in nocodazole-treated cells expressing HEC1 variants as indicated. Cells in (**f**) were treated with an additional 125 nM dose of reversine before mitotic entry. Data are the average of three experiments (solid line)±s.e.m. (transparent area) for at least 78 cells in (**e**) and 81 cells in (**f**). (**g**) Quantification of time-lapse analysis of nocodazole-arrested cells treated with an additional 250 nM dose of reversine after mitotic entry. Representation as in (**e**,**f**). At least 92 cells were followed per mutant. Scale bars, 5 μm.

To inhibit biorientation, we prevented spindle bipolarization by treating cells with the Eg5 inhibitor S-trityl-L-cysteine (STLC) (ref. 38). As expected, STLC-treated cells expressing HEC1-Wildtype (HEC1-WT), -9D and -Δ80 accumulated a marker for unattached chromosomes, MAD2, at their kinetochores, due to either frequent destabilization of kinetochore–microtubule interactions by the Aurora B kinase (HEC1-WT) or inherently low affinity of kinetochores for microtubules (HEC1-9D and -Δ80; Fig. 1a–d). As a result, the SAC was persistently activated in these cells, as evidenced by time-lapse imaging (Fig. 1e). In contrast, monopolar HEC1-9A-expressing cells were able to form stable kinetochore–microtubule attachments, as shown by the presence of cold-resistant microtubules (Supplementary Fig. 2a,b) and loss of MAD2 from virtually all kinetochores (Fig. 1b–d). This was not due to diminished Aurora B activity, as various proteins that rely on Aurora B for kinetochore localization, including MAD2, BUB1 and BUBR1 (ref. 29), bound kinetochores when microtubules were depolymerized by nocodazole, with equal efficiency as HEC1-WT cells (Fig. 2a–d; Supplementary Fig. 4a,b). Strikingly, the vast majority of monopolar HEC1-9A-expressing cells exited mitosis within hours of nuclear envelope breakdown, showing that the SAC had been satisfied in these cells (Fig. 1e). Accordingly, phosphorylation of the SAC scaffold KNL1 on one of its MELT motifs was undetectable at kinetochores of monopolar HEC1-9A-expressing cells, illustrating diminished signalling by the critical SAC kinase MPS1 (Supplementary Fig. 2c,d), a hallmark of a silenced SAC (ref. 39). To rule out that differences in ectopic HEC1 expression and/or HEC1 depletion, obscured in bulk analyses such as immunoblotting, could account for different SAC responses in our time-lapse experiments, we directly correlated GFP-HEC1 kinetochore levels to duration of mitosis by single-cell analyses. As seen in Fig. 1f,g, monopolar HEC1-9A cells exited mitosis within 100 min, while all cells expressing HEC1-WT at kinetochores to levels comparable to HEC1-9A remained arrested for the duration of the experiment. Moreover, expression of HEC1-9A while retaining endogenous HEC1 resulted in a similar, albeit less severe phenotype (Supplementary Fig. 3a), showing a dominant effect of HEC1-9A on SAC silencing. Similar data were obtained in non-transformed RPE-1 cells (Supplementary Fig. 3b–e).

HEC1-9A binds microtubules tighter than metaphase HEC1-WT (refs 35, 40, 41) and this could conceivably affect SAC silencing in unnatural ways. To exclude this, we constructed cell lines expressing HEC1-8A/S15D or -8A/S55D, two recently described HEC1-8A/1D mutants that have microtubule-binding properties similar to those of HEC1-WT (refs 35, 40, 41) but are, like HEC1-9A, refractory to Aurora B activity (Supplementary Fig. 2f). When forced to form only non-bioriented attachments, cells expressing either of these mutants exited mitosis with a rate close to that of HEC1-9A-expressing cells (Supplementary Fig. 2g,h).

Although HEC1-9A cells were not significantly slower in progressing through mitosis than HEC1-WT cells (Supplementary Fig. 2e), monopolar HEC1-9A cells exited more slowly than their bipolar counterparts (Fig. 1e versus Supplementary Fig. 2e). Analysis of the number of MAD2-positive kinetochores in monopolar HEC1-9A cells over time showed that these kinetochores progressively obtained stable attachments, and the timing of removal of MAD2 from all kinetochores correlated well with observed exit rates (Fig. 1h,i, compare with Fig. 1e: ∼50% had exited mitosis at 120 min when ∼50% of the cells had no more detectable MAD2 at their kinetochores). The relatively slow exit of monopolar HEC1-9A cells is thus likely due to slow microtubule capture, possibly because of unfavourable orientation of the unattached kinetochore of a monotelic attached pair (Arrowheads in Fig. 1h). These results were confirmed with live imaging of single cells constitutively expressing fluorescent-tagged-MAD2: monopolar HEC1-WT cells maintained multiple MAD2-positive kinetochores in mitosis when filmed ∼30 min after mitotic entry, while HEC1-9A cells had 1-2 MAD2-positive kinetochores that became attached (Fig. 1j). Together, these data show SAC silencing in cells with stably attached, non-bioriented kinetochores.

### The SAC is fully proficient in HEC1-9A-expressing cells

**Figure 3:**
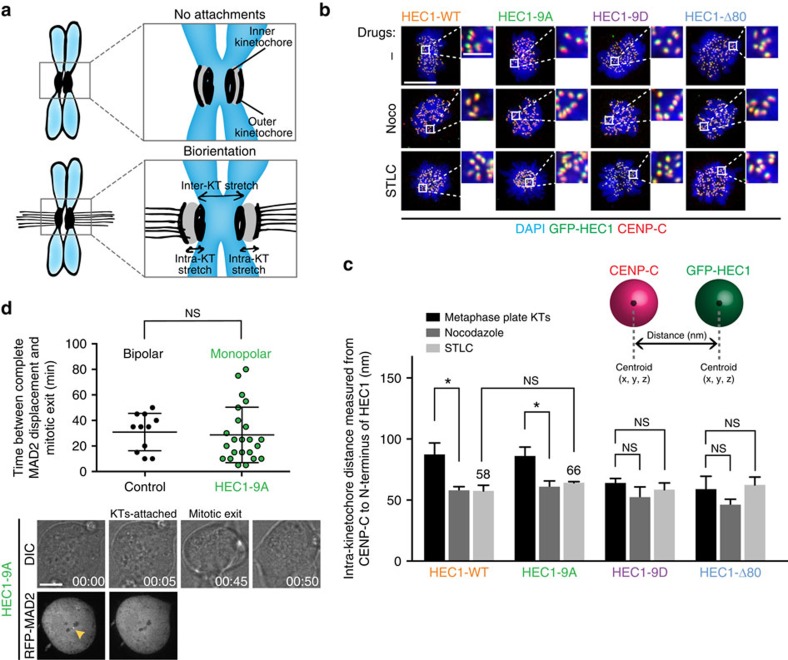
Full intra-kinetochore stretch is not required for SAC silencing in monopolar HEC1-9A cells. (**a**) Cartoons illustrating the concept of kinetochore distances in attached or unattached states. Relative to unattached chromosomes (upper drawing), sister kinetochores of bioriented chromosomes (bottom drawing) are positioned further away from one another and experience stretch within individual kinetochores. (**b**,**c**) Representative images (**b**) and quantification (**c**; average of three experiments±s.e.m.) of the distance between HEC1(N) and CENP-C in fixed cells (illustration in **c**). Cells were treated with nocodazole or STLC before mitotic entry, after which they were treated with MG132 for 2 h before fixation. At least 147 kinetochores were measured for each mutant. Scale bar, 5 μm. Scale bar inset, 1 μm (**d**) Quantification (upper panel) and representative images (lower panel) of time-lapse single-cell analysis of bipolar control cells or monopolar HEC1-9A cells expressing RFP-MAD2. Cells were released from RO and imaging was initiated after mitotic entry. Note that MAD2 is overexpressed in these cells. Data are the average of two experiments±s.d. Quantifications were subjected to unpaired Student's *t*-test against the values measured for control cells. *P<0.01. Scale bar, 10 μm.

Some recent studies hinted at a role for HEC1 tail phosphorylation in the SAC (refs 42, 43), while others and we have shown that MPS1 localization and SAC activity are normal in cells expressing HEC1-Δ80 (refs 34, 35, 44). We thus wished to verify that the SAC was fully functional in cells expressing HEC1-9A, especially because a weakened SAC could potentially have difficulty preventing mitotic exit when only few kinetochores are signalling. The following analyses showed that the SAC in our HEC1-9A cells was maximally proficient. First, the SAC proteins MAD2, BUBR1 and BUB1 localized at normal levels to kinetochores of nocodazole-treated cells, independent of the expressed HEC1 variant (Fig. 2a–d; Supplementary Fig. 4a,b). Second, all cells maintained long mitotic arrests when spindle microtubules were depolymerized by nocodazole (Fig. 2e). Third, a similar long arrest in nocodazole was maintained by all cell lines when the SAC was artificially weakened by addition of a low dose of the MPS1 inhibitor reversine, previously used by us and others to uncover subtle SAC deficiencies including those as a result of incomplete HEC1 depletion (Fig. 2f)^24^,^25^,^45^. Fourth, similar exit rates were observed for HEC1-WT and HEC1-9A-expressing cells after nocodazole-arrested cells were forced to exit mitosis by addition of reversine, showing that efficiency of SAC silencing was unaffected (Fig. 2g). Finally, MAD2-positive kinetochores in STLC-treated HEC1-9A cells recruited similar levels of MAD2 as those of HEC1-WT cells (Supplementary Fig. 4c,d). Since MAD2 levels correlate with strength of the SAC signal^46^, this showed HEC1-9A kinetochores are capable of maximal SAC signalling when their kinetochores are unattached. This was further supported by our observation that monopolar HEC1-9A cells (23/23 cells) exited mitosis only after attachment of all kinetochores (Fig. 3d).

### Full kinetochore stretch is not essential for SAC silencing

Individual kinetochores deform upon microtubule attachment; the distance between inner- and outer-kinetochore components increases as a result of forces imposed by dynamic microtubules (Fig. 3a)^17^,^47^,^48^. This phenomenon, referred to as intra-kinetochore stretching, has been correlated to mitotic exit, and as such has been put forth as the primary signal that satisfies the SAC (refs 17, 18). To examine if SAC silencing in monopolar HEC1-9A cells correlated with intra-kinetochore stretching, we measured the distance between the inner- and outer-kinetochore in our cell lines, using antibodies against CENP-C and the N-terminus of HEC1 (GFP; HEC1(N); see cartoon in Fig. 3c and see Supplementary Methods for details on method and the various technical controls). As a control, we measured the CENP-C to HEC1(N) distance of unattached chromosomes (nocodazole-treated cells) and of bioriented and congressed chromosomes (in MG132-treated cells). In agreement with published measurements^17^,^48^, we observed an increased distance of ∼25 nm between CENP-C and HEC1(N) of both HEC1-WT and -9 A-expressing cells when kinetochores of bioriented chromosomes were compared with those of unattached ones (Fig. 3b). Unexpectedly, we observed only a small, non-significant difference between CENP-C and HEC1(N) in monopolar HEC1-9A-expressing cells when compared with HEC1-WT monopoles (Fig. 3b,c), despite removal of MAD2 (Fig. 1b–d) and SAC silencing (Fig. 1e). Furthermore, inhibiting microtubule dynamics in monopolar cells by addition of 1 μM Taxol^17^ did not prevent mitotic exit of the HEC1-9A cells (Supplementary Fig. 5). These data show that the SAC can be satisfied without substantial intra-kinetochore stretch. We furthermore found no evidence of a role for such stretch in the efficiency of SAC silencing: Monopolar HEC1-9A cells did not take significantly longer than bipolar control cells to exit mitosis after attachment of the final kinetochore (Fig. 3d). These observations imply that microtubule attachment *per se* is sufficient to silence the SAC and that full intra-kinetochore stretch and pulling forces from microtubules are not a prerequisite.

## Discussion

Here we have shown that formation of stable kinetochore–microtubule attachments, irrespective of kinetochore orientation and stretching, is sufficient to satisfy the SAC in human cells. Although full intra-kinetochore stretch is not required for SAC silencing in our system, we cannot rule out that the small, statistically insignificant ∼8 nm increase in the distance between CENP-C and HEC1(N) that we observed in monopolar HEC1-9A compared with HEC1-WT promotes SAC silencing or that more significant stretch occurs between proteins other than those measured. Given the presented evidence, however, we favour the interpretation that stretch may not play a significant role, at least in human cells. This interpretation is perhaps not universally applicable to all eukaryotes, as putting distance between Mps1 and Spc105 was recently proposed to be a mechanism for SAC silencing in *Saccharomyces cerevisiae*^49^.

What then, if any, is the role of biorientation in SAC silencing? We propose that tension, either within kinetochores or between kinetochores, promotes SAC silencing indirectly by promoting stabilization of kinetochore–microtubule attachment^16^. Stable microtubule binding in turn inhibits SAC signalling in a number of ways. Microtubules promote dynein-dependent stripping of Spindly and/or other SAC components^50^,^51^,^52^, and directly displace the critical SAC kinase MPS1 (refs 42, 53). Microtubule engagement could in addition promote biochemical changes in the kinetochore that initiate SAC silencing, such as those elicited by the SAC silencing phosphatases PP1 and PP2A-B56 (refs 39, 54). In agreement with the possibilities that microtubules regulate the balance of kinase/phosphatase signalling at kinetochores is our observation that the PP1 target KNL1 is efficiently dephosphorylated in monopolar HEC1-9A cells. Our ability to uncouple stable attachment from biorientation now provides the tool to interrogate the various ways in which microtubules impact on the SAC signalling system.

## Methods

### Cell culture and transfection

HeLa and RPE1 FlpIn cells were respectively grown in DMEM and DMEM/F12 supplemented with 8% FBS (Lonza), penicillin/streptomycin (50 μg ml^−1^), Ultra-glutamine (Sigma; 2 mM), blasticidin (4 μg ml^−1^) and hygromycin for HeLa (200 μg ml^−1^) or puromycin for RPE1 (1.6 μg ml^−1^). 293Ts were grown in DMEM supplemented with 8% FBS (Lonza), penicillin/streptomycin (50 μg ml^−1^) and Ultra-glutamine (Sigma; 2 mM). Plasmids were transfected using Fugene HD (Roche) for HeLa or Lypofectamin LTX (Invitrogen) for RPE1 according to the manufacturer's instructions. To generate stably integrated HeLa and RPE1 FlpIn cell lines, pCDNA5-constructs were co-transfected with pOG44 recombinase in a 1:9 for HeLa and 1:5 ratio for RPE1 (ref. 55). Constructs were expressed by addition of 1 μg ml^−1^ doxycycline for 24 h siHEC1 (custom; Thermo Fisher Scientific; 5′-CCCUGGGUCGUGUCAGGAA-3′) and siGAPDH (Thermo Fisher Scientific; D-001830-01-50) were transfected using HiPerfect (Qiagen) according to manufacturer's instructions.

Cells expressing RFP-MAD2 were obtained through lentiviral transduction and subsequent selection with puromycin (1.6 μg ml^−1^).

### Plasmids

pCDNA5-pEGFP-HEC1 constructs and cloning strategies are described in refs 31, 34. For generation of stable RPEs, the HEC1 variants were subcloned to pCDNA5-pEGFPAID-puro (a kind gift from Andrew Holland^56^) with SnaBI/ApaI. tagRFP-MAD2 was PCR-ed with primers overlapping pLV-CMV (lentiviral plasmid containing the CMV promoter) and inserted using the Gibson Assembly strategy^57^.

### Knockdown and reconstitution of HEC1

To knockdown and reconstitute HEC1 in HeLa-FLpIn cell lines, cells were transfected with 120 nM HEC1 or mock siRNA for 16 h after which cells were arrested in early S phase for 24 h by addition of thymidine (2 mM). Subsequently, cells were released from thymidine and transfected again with 40 nM siRNA. Around 8–10 h after the release, cells were treated with doxycycline (1 μg ml^−1^) and arrested for a second time in S phase for 14–16 h. Finally, cells were released from thymidine, treated with the indicated drugs (STLC at 20 μM, nocodazole at 3.3 μM, Taxol at 1 μM, reversine at 125 nM, RO at 5 μM) and used for experiments. For immunofluorescence imaging, cells were treated with proteasome-inhibitor MG132 (5 μM) for 120 min before fixation.

### Live-cell imaging and immunofluorescence

Live imaging of single cells and H2B-mCherry-expressing cells was performed on a personal DeltaVision system (Applied Precision/GE Healthcare) equipped with a Coolsnap HQ2 CCD camera (Photometrics) and Insight solid-state illumination (Applied Precision/GE Healthcare). Cells were plated in 8-well plates (μ-Slide 8 well, Ibidi), treated as described above and imaged in a heated chamber (37 °C and 5% CO^2^) using a × 60/1.42 numerical aperture (NA) or × 100/1.4 NA UPlanSApo objective (Olympus) at 2 × 2 binning. Images were acquired every 4 min and deconvolved using standard settings in SoftWorx (Applied Precision/GE Healthcare) software. Multiple z layers with 0.20 μm intervals were acquired and projected to a single layer by maximum intensity projection. Differential interference contrast (DIC) microscopy images were single layed. Images for intra-kinetochore measurements were acquired with 0.10 μm intervals.

Live imaging of single-cells-expressing RFP-MAD2 was performed on a Leica DMI6000 Ultraview VoX Spinning Disk Microscope (PerkinElmer). Images were acquired every 3–5 min with a Hamamatsu Orca R2 camera using a × 100/1.4 NA objective and Velocity 3D Image Analysis Software. Multiple z layers with 1 μm intervals were acquired and DIC images were obtained as a reference.

For other live-cell imaging experiments, cells were plated in 24-well plates (Corning Incorporated), and subjected to DIC microscopy on an Olympus IX81 inverted microscope equipped with a × 10/0.30 NA CPlanFLN objective lens (Olympus), Hamamatsu ORCA-ER camera and processed by Cell̂M software (Olympus). The cells were kept in a heated chamber (37 °C and 5% CO^2^) and images were acquired every 4 min at 2 × 2 binning. For fluorescent imaging of H2B, cells were transduced 24 h before imaging with Baculovirus carrying H2B-mCherry under the control of a CMV promoter.

For fixed cell immunofluorescence microscopy, cells plated on round 12-mm coverslips (No. 1.5) were pre-extracted with 37 °C 0.1% Triton X-100 in PEM (100 mM Pipes (pH 6.8), 1 mM MgCl_2_ and 5 mM EGTA) for ±45 s before fixation (with 4% paraformaldehyde, 0.1% Triton X-100, 100 mM Pipes, pH 6.8, 1 mM MgCl_2_, and 5 mM EGTA) for 5–10 min. For cold-shock experiments, cells were placed on ice and treated with ice-cold media for 9 min before pre-extraction and fixation. Coverslips were washed twice with cold PBS and blocked with 3% BSA in PBS for 16 h at 4 °C, incubated with primary antibodies for 16 h at 4 °C, washed 4 times with PBS containing 0.1% Triton X-100, and incubated with secondary antibodies for an additional hour at room temperature. Coverslips were then washed twice with PBS/0.1% Triton X-100, incubated with DAPI for 2 min, washed again twice with PBS and mounted using Prolong Gold antifade (Molecular Probes). All images were acquired on a deconvolution system (DeltaVision Elite; Applied Precision/GE Healthcare) with a × 100/1.40 NA UPlanSApo objective (Olympus) using SoftWorx 6.0 software (Applied Precision/GE Healthcare). Images are maximum intensity projections of deconvolved stacks. Images of cold-shock experiments are sum projection images.

### Image quantification

Analysis of live-cell imaging experiments was carried out with ImageJ software. Time in mitosis was defined as the time between nuclear envelope breakdown and anaphase-onset or cell flattening.

For quantification of immunostainings, all images of similarly stained experiments were acquired with identical illumination settings. Cells expressing comparable levels of exogenous protein were selected for analysis and analysed using ImageJ. An ImageJ macro was used to threshold and select all centromeres and all chromosome areas (excluding centromeres) using the DAPI and CENP-C. This was used to calculate the relative average kinetochore intensity of various proteins ((centromeres–chromosome arm intensity (kinetochore localized protein of interest))/(centromeres–chromosome arm intensity (CENP-C))). A similar method was used to measure total spindle tubulin after cold-shock experiments.

### Intra-kinetochore distance measurements

Cells were treated, fixed, stained and imaged as described above. Analysis was performed with the ImageJ plugin ‘Object counter 3D' on deconvolved, non-projected images. In this manner, coordinates (*x*, *y* and *z*) of the center of mass of the GFP (-HEC1) and CENP-C signals were identified and used accordingly to calculate the distance between the inner (CENP-C) and outer kinetochore (GFP) independent of direction of kinetochore stretching. To correct for chromatic aberrations, GFP was stained with two secondary antibodies with different fluorophores in each individual cell line. For each experiment, the distance between the two fluorophores was measured for 25 kinetochores in 5–7 cells, averaged and used to correct the position of the CENP-C signal in the intra-kinetochore measurements.

### Immunoblotting

Cells were treated as described above and entered mitosis in the presence of nocodazole. Mitotic cells were isolated by mitotic shake off and lysed in Laemmli lysis buffer (4% SDS, 120 mM Tris (pH 6.8) and 20% glycerol). Lysates were processed for SDS–polyacrylamide gel electrophoresis and transferred to nitrocellulose membranes for immunoblotting. Immunoblotting was performed using standard protocols. Visualization of signals was performed on a scanner (Amersham Imager 600) using enhanced chemiluminescense.

### Antibodies

The following primary antibodies were used for immunofluorescence imaging: CENP-C (polyclonal guinea pig, 1:2,000; MBL Catalog#: PD 030), α-TUBULIN (mouse monoclonal, 1:10,000; Sigma-Aldrich Catalog#: T5168), γ-TUBULIN (rabbit polyclonal, 1:500; Sigma-Aldrich Catalog#: T5192), HEC1 (mouse monoclonal 9G3, 1:2,000; Abcam Catalog#: Ab-3613), GFP (custom rabbit polyclonal raised against full-length GFP as antigen^58^, 1:10,000), GFP (mouse monoclonal, 1:1,000; Roche Catalog#: 12-814-460-001), MAD2 (custom rabbit polyclonal raised against full-length 6 × His-tagged MAD2 as antigen^59^, 1:2,000), BUBR1 (rabbit polyclonal, 1:1,000; Bethyl Catalog#: A300-386 A) and BUB1 (rabbit polyclonal, 1:1,000; Bethyl Catalog#: A300-373 A-1).

Secondary antibodies (Invitrogen Molecular Probes, all used at 1:600) were highly crossed absorbed goat anti-guinea pig Alexa Fluor 488 (Catalog#: A11073) and 647 (Catalog#: A21450), goat anti–rabbit Alexa Fluor 488 (Catalog#: A11034), 568 (Catalog#: A11036) and 647 (Catalog#: A21245), and anti–mouse Alexa Fluor 488 (Catalog#: A11029) and 568 (Catalog#: A11031).

## Additional information

**How to cite this article:** Etemad, B. *et al*. Kinetochore–microtubule attachment is sufficient to satisfy the human spindle assembly checkpoint. *Nat. Commun.* 6:8987 doi: 10.1038/ncomms9987 (2015).

## Supplementary Material

Supplementary Information Supplementary Figures 1-5
