# Supplementary material for: Kinetochore–microtubule attachment is sufficient to satisfy the human spindle assembly checkpoint
Source: Nat Commun. 2015 Dec 1;6:8987. doi: 10.1038/ncomms9987 (PMC4686852; doi:10.1038/ncomms9987)
Supplement: Supplementary Information — Supplementary Figures 1-5 [file ncomms9987-s1.pdf]

Etemad et al., Supplementary Fig. 1

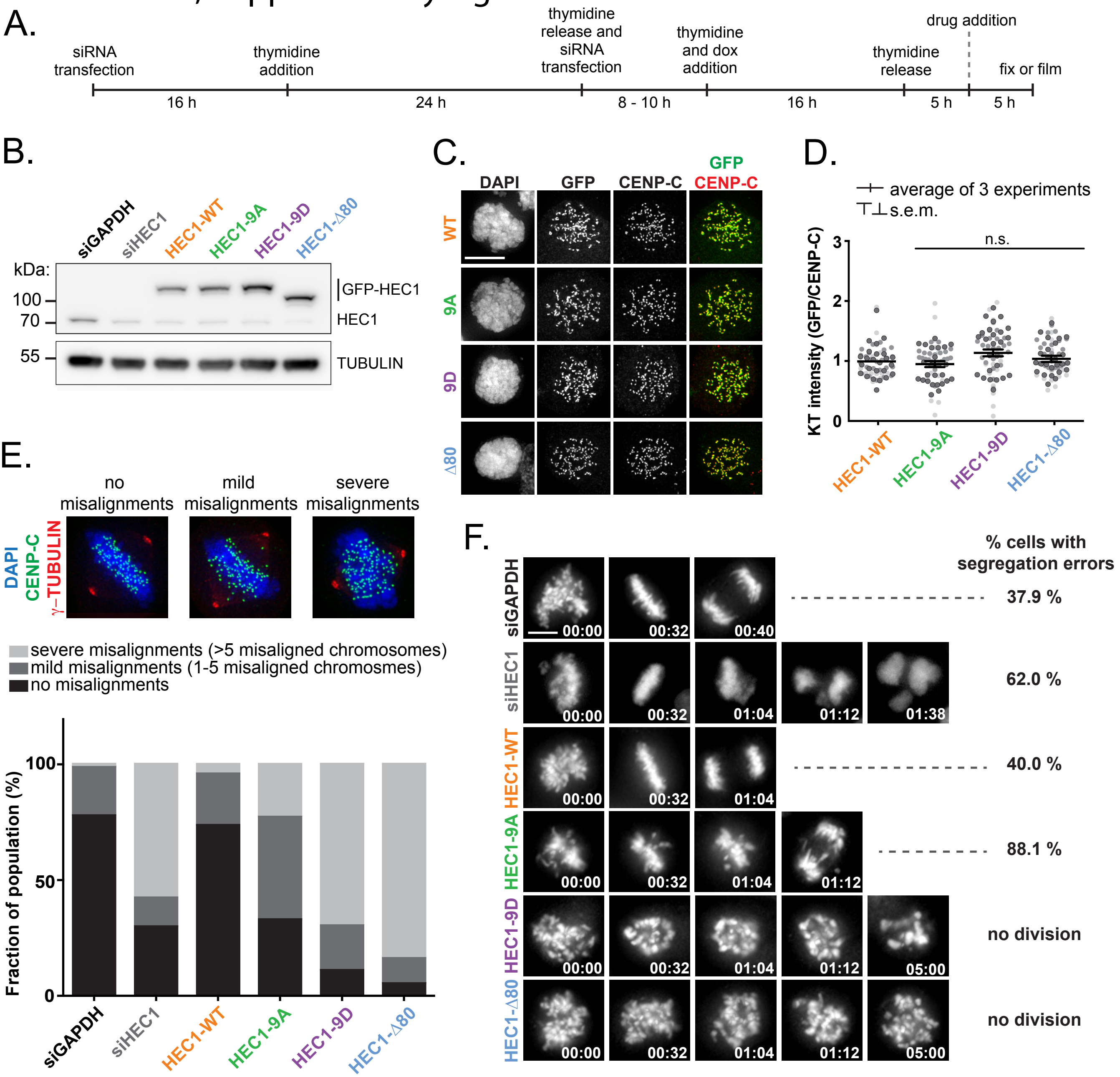

Supplementary Fig. 1. Characterization of cells expressing variants of HEC1. (**a**) Scheme of workflow used throughout this paper. Exceptions are indicated. (**b**) Immunoblot showing HEC1 knock down and expression of siRNA-resistant GFP-tagged versions of HEC1 in a mitotic population. (**c**) Immunofluorescent labeling of indicated proteins in nocodazole-arrested cells. Channel colors of merged images match those of the labels. (**d**) Quantification of the kinetochore intensity of GFP in (c) normalized to the kinetochore intensity of CENP-C. Each dot represents one cell. The data points of three independent experiments are depicted in different shades of gray. Also shown is the average fold-change of three experiments (± s.e.m.) normalized to the average values of HEC1-WT. A total of at least 63 cells were measured per mutant. For statistical analysis, an unpaired Student's *t*-test was performed against the measured values of HEC1-WT expressing cells. **P* < 0.01. (**e**) Alignment assay of cells expressing mutant versions of HEC1 as indicated. Top panel shows examples of the scored categories. Aligment status was scored only in cells with centrosomes that lay in the same plane. (**f**) Representative stills from movies of cells co-expressing variants of HEC1 and mCherry-H2B. At least 50 cells were scored per condition in two independent experiments. Scale bars, 5 µm.

Etemad *et al*., Supplementary Fig. 2

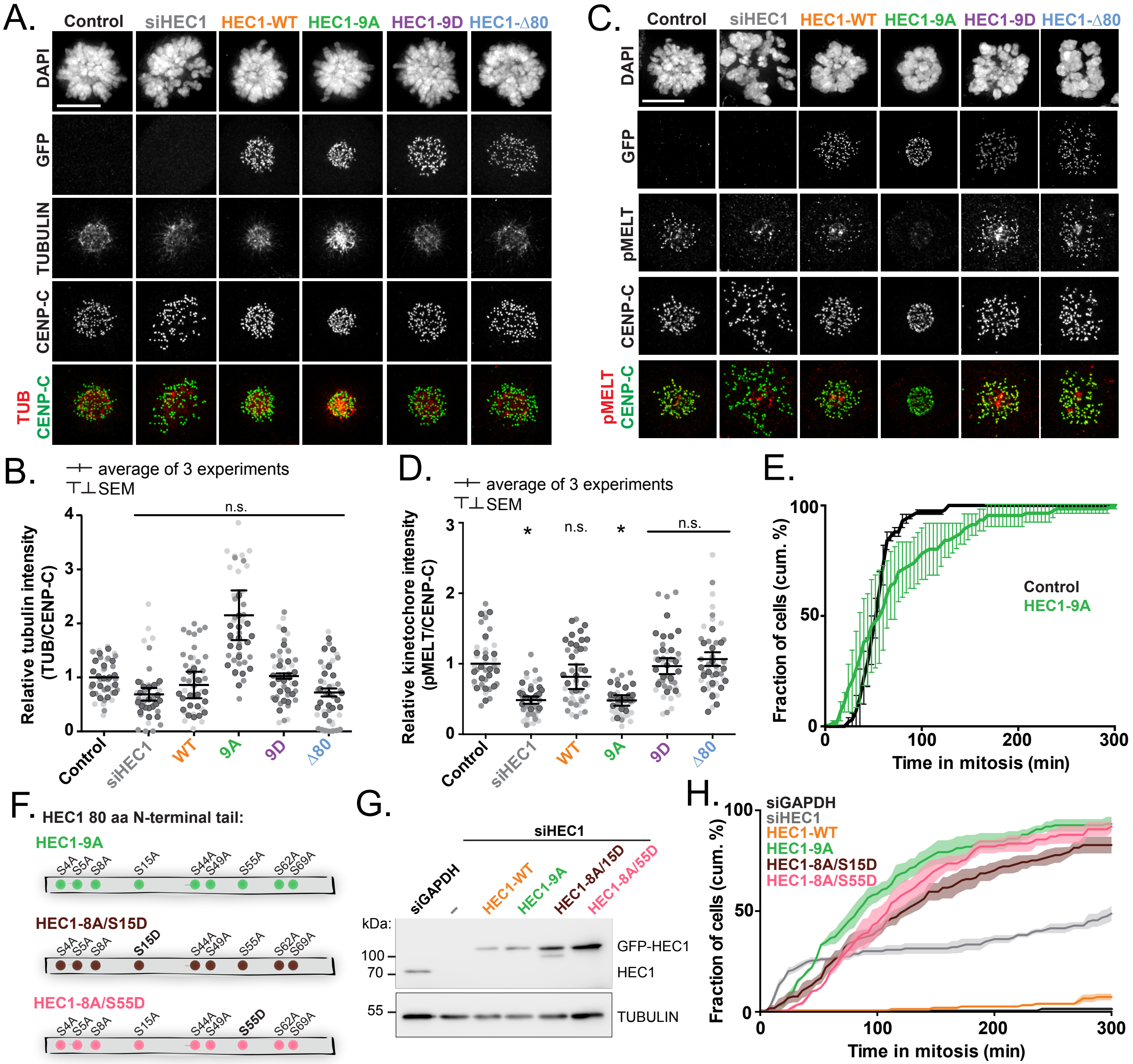

Supplementary Fig. 2. Characterization of attachments in monopolar HEC1-9A expressing cells. (**a-d**) Immunofluorescent labeling of indicated proteins in STLC-arrested cells (a, c) and quantification thereof (b, d). To visualize stable microtubules in (a, b) cells were cold-treated prior to fixation. Channel colors of merged images match those of the labels. Quantification is normalized to the kinetochore intensity of CENP-C and is the average fold-change of three experiments (± s.e.m.) normalized to the values of control cells. Each dot represents one cell. The data points of three independent experiments are depicted in different shades of gray. A total of at least 45 cells were measured per mutant in (b) and 47 in (d).(**e**) Time-lapse analysis of control and HEC1-9A expressing cells going through mitosis with bipolar spindles. Data are the average of two experiments ± s.e.m. (error bars) for at least 97 cells. (**f**) Cartoon of the N-terminal tail of HEC1 showing the mutated residues in the indicated variants of HEC1. (**g**) Immunoblot showing HEC1 knock down and expression of siRNA-resistant GFP-tagged versions of HEC1. (**h**) Time-lapse analysis of cells expressing indicated HEC1 mutants. Cells entered mitosis in the presence of STLC. Data are the average of three experiments (solid line), ± s.e.m. (transparent area) for at least 130 cells. All quantifications were subjected to unpaired Student's *t*-test against the measured values of siGAPDH-transfected cells. *$P < 0.01$. Scale bar, 5 μm.

Etemad *et al*., Supplementary Fig. 3

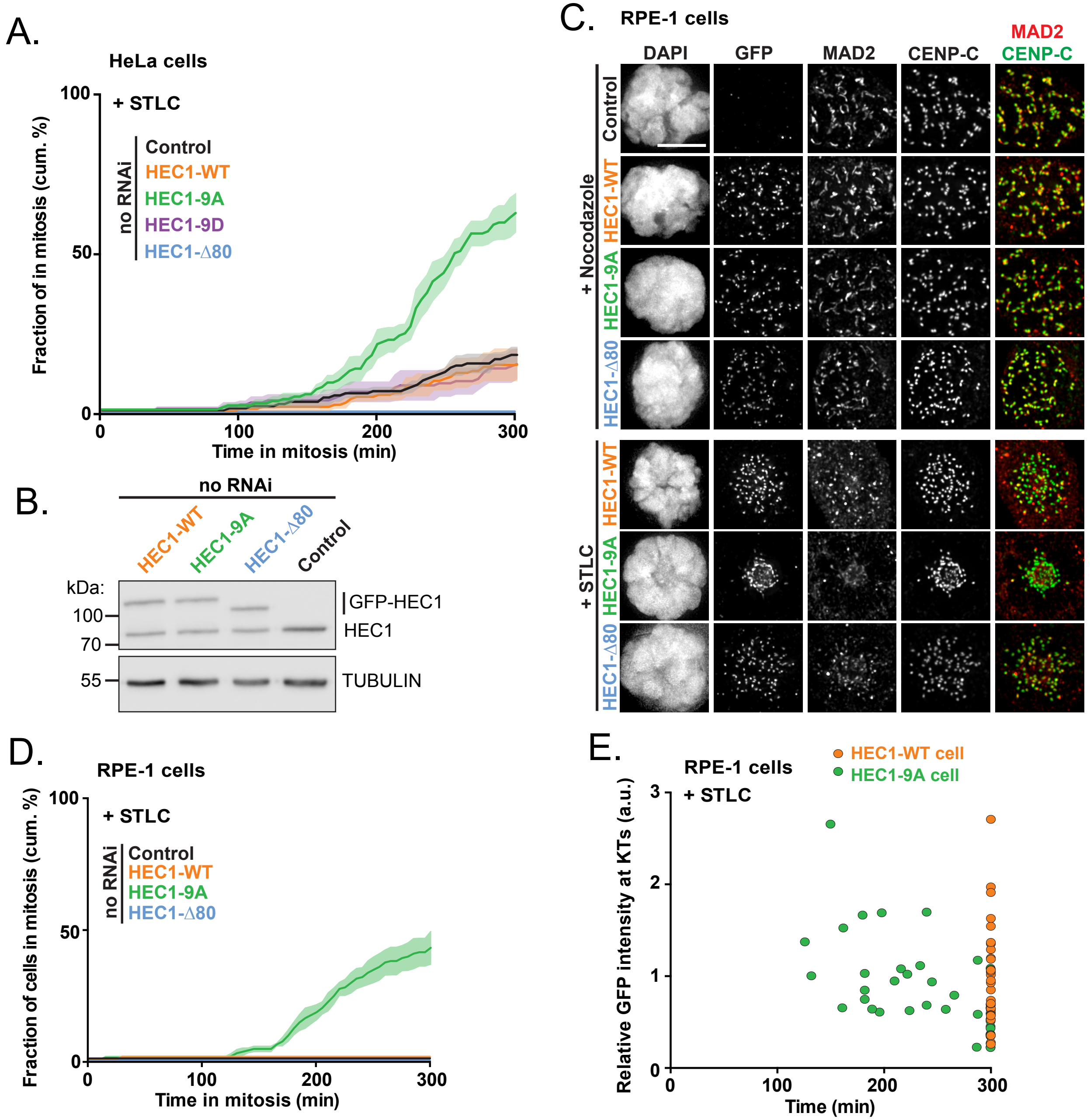

Supplementary Fig. 3. Characterization of HEC1-9A expressing RPE-1 cells. (**a**) Time-lapse analysis of duration of mitotic arrest in STLC-treated HeLa cells overexpressing HEC1 variants as indicated. (**b**) Immunoblot showing HEC1 expression in a mitotic population of RPE-1 cells. Lower band represents endogenous HEC1, upper one is the tagged mutant version. (**c**) Immunofluorescent labeling of indicated proteins in nocodazole- and STLC-arrested cells. Cells were treated with MG132 for 2 hours prior to fixation. Channel colors of merged images match those of the labels. Scale bar, 5 μm. (**d**) Time-lapse analysis of duration of mitotic arrest in STLC-treated RPE-1 cells overexpressing HEC1 variants. (**e**) Time-lapse single cell analysis of HEC1-WT and HEC1-9A expressing RPE-1 treated with STLC plotted against the total GFP-kinetochore levels. The total GFP-kinetochore level of analyzed cells was measured at mitotic entry and normalized against the average level measured in HEC1-WT cells. Data are from two independent experiments. Each dot represents one cell. For HEC1-WT 34 cells were imaged, for HEC1-9A 42 cells. Data in (a) and (d) are the average of three experiments (solid line) ± s.e.m. (transparant area) for at least 144 cells in (a) and 180 cells in (d).

Etemad *et al*., Supplementary Fig. 4

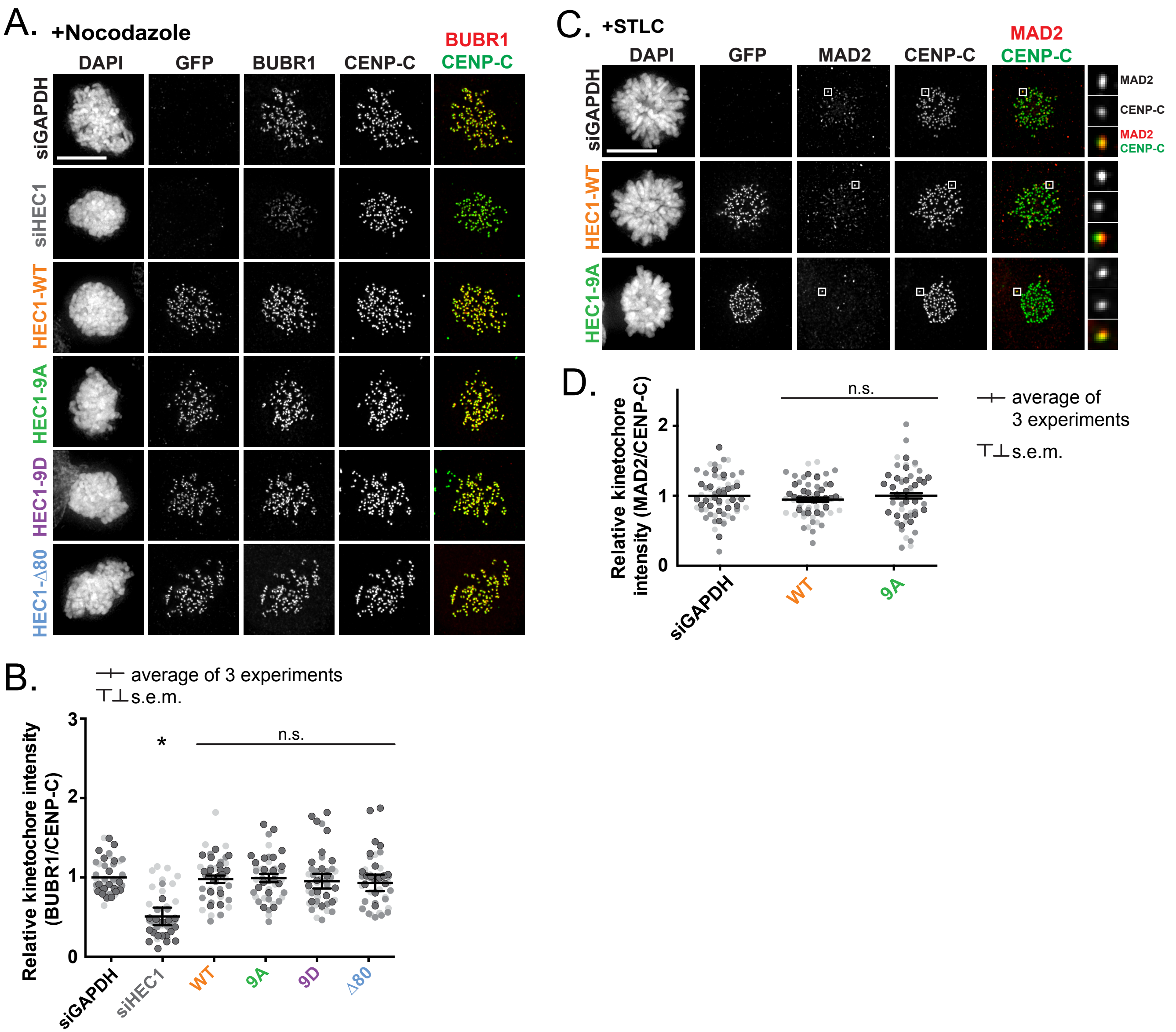

Supplementary Fig. 4. SAC protein levels of cells expressing HEC1 variants. (**a-d**) Immunofluorescent labeling of indicated proteins in nocodazole- (a, b) and STLC-treated cells (c, d). Channel colors of merged images match those of the labels. In (c, d) cells entered in the presence of STLC and were subsequently treated with MG132 for two hours prior to fixation. MAD2 and CENP-C levels were measured on kinetochores positive for this protein.

Quantification is normalized to the kinetochore intensity of CENP-C and is the average fold-change of three experiments (± s.e.m.) normalized to the values of control cells. The data points of three independent experiments are depicted in different shades of gray. A total of at least 50 cells was measured per mutant in (b) and 57 kinetochores in (d). Quantifications were subjected to unpaired Student's *t*-test against the measured values of siGAPDH-transfected cells. *$P < 0.01$. Scale bars, 5 µm.

Etemad *et al.*, Supplementary Fig. 5

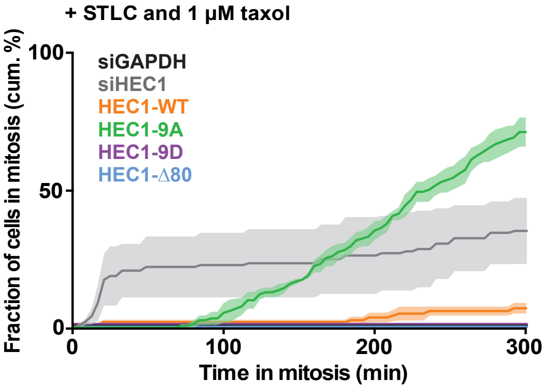

Supplementary Fig. 5. Microtubule based pulling forces have no role in SAC silencing in HEC1-9A cells. Time-lapse analysis of duration of mitotic arrest in cells treated with STLC and Taxol prior to mitotic entry. Data are average of three experiments (solid line) ± s.e.m. (transparant area) for at least 100 cells per mutants.
